# A Solution to the Challenge of Optimization on ''Golf-Course''-Like Fitness Landscapes

**DOI:** 10.1371/journal.pone.0078401

**Published:** 2013-11-05

**Authors:** Hygor Piaget M. Melo, Alexander Franks, André A. Moreira, Daniel Diermeier, José S. Andrade, Luís A. N. u. n. e. s. Amaral

**Affiliations:** 1 Departamento de Física, Universidade Federal do Ceará, Fortaleza, Ceará, Brazil; 2 Department of Chemical and Biological Engineering, Northwestern University, Evanston, Illinois, United States of America; 3 Department of Statistics, Harvard University, Cambridge, Massachusetts, United States of America; 4 MEDS, Kellogg School of Management, Northwestern University, Evanston, Illinois, United States of America; 5 Northwestern Institute on Complex Systems, Northwestern University, Evanston, Illinois, United States of America; 6 Canadian Institute for Advanced Research, Toronto, Ontario, Canada; 7 Howard Hughes Medical Institute, Northwestern University, Evanston, Illinois, United States of America; Swiss Federal Institute of Technology (ETH Zurich), Switzerland

## Abstract

Genetic algorithms (GAs) have been used to find efficient solutions to numerous fundamental and applied problems. While GAs are a robust and flexible approach to solve complex problems, there are some situations under which they perform poorly. Here, we introduce a genetic algorithm approach that is able to solve complex tasks plagued by so-called ''golf-course''-like fitness landscapes. Our approach, which we denote *variable environment genetic algorithms* (VEGAs), is able to find highly efficient solutions by inducing environmental changes that require more complex solutions and thus creating an evolutionary drive. Using the density classification task, a paradigmatic computer science problem, as a case study, we show that more complex rules that preserve information about the solution to simpler tasks can adapt to more challenging environments. Interestingly, we find that conservative strategies, which have a bias toward the current state, evolve naturally as a highly efficient solution to the density classification task under noisy conditions.

## Introduction

Natural evolution has a demonstrated ability to solve complex problems and to build on existing solutions. The power of the evolutionary approach inspired a number of computer scientists to study evolutionary systems culminating in the invention of genetic algorithms (GAs) by John Holland (see Mitchell [1] for a brief history). Holland's work [2], set the stage for much of the later studies of the foundations of GA and related evolutionary computation approaches.

In spite of their success and of the on-going work on determining the best encoding of a candidate solution into a chromosome or the best set of parameter values for population size, selection of reproducing individuals, and crossover, inversion and mutation rates [4], there are still many situations under which simple GAs fails to find good solutions. To understand why, recall that GAs explores a given search space — the set of all possible candidate solutions — trying to optimize the fitnesses of the evolving individuals in the population.

The idea that some optimization problems may not be addressed with simple GAs was formalized with the so called ''no free lunch'' theorems [5]. Specifically, difficulties arise when the fitness landscape (i) is too rugged, in which case the numerous local fitness maxima make it difficult to discover the global maximum and may lead to trapping in a non-optimal solution, or (ii) is too flat — the so-called ''golf-course-like'' landscape [6] — in which case the lack of a significant gradient in the fitness landscape means that there is no driving bias toward fitness maxima. While heuristic strategies for addressing the former are well known, strategies for tackling the latter are still being actively investigated.

Different approaches have been proposed to improve on classical GAs, including co-evolution [7], dynamic environments [8] and incremental complexification [9]. The challenge is that if the fitness landscape is flat, fitness selection is blind. If, additionally, the density of maxima is very low, then one would need an extremely large population size in order to have a non-negligible chance that at least one individual has a genome close to a fitness maximum.

In order to address the challenges posed by phenomena characterized by golf-course-like fitness landscape, we draw inspiration from nature. Specifically, we note that the evolution of complex traits has occurred in steps. For example, the evolution of the eye — i.e. a structure able to convert electromagnetic radiation into information about the surrounding environment — did not occur in a single event [10]. The first proto-eyes were likely only able to detect changes in light intensity. In the same spirit, we propose that in order to obtain solutions to problems characterized by golf-course-like fitness landscapes one must first solve simpler, but related, problems.

Additionally, we note that natural evolution appears to occur faster at transition zones between different environments. Indeed, numerous studies show that bacteria can develop antibiotic resistance faster in the presence of concentration gradients [11]–[14]. In the same spirit, we propose the use of varying environments to evolve populations highly fit for challenging environments.

### Density Classification

As a case study, we consider here the density classification task [15] in noisy environments [16]. Density classification is a paradigmatic information aggregation problem [1]: the agents in the population initially hold information in the form of a binary value. The goal is for all agents to reach a consensus on the binary value that was initially in the majority, while only sharing information with a ''small'' number of neighbors.

**Figure 1:**
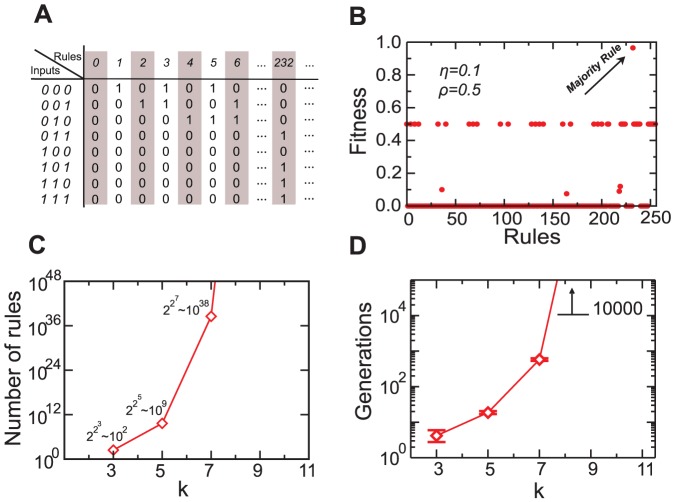
The evolutionary challenge posed by golf-course-like fitness landscapes. (A) For the density classification task, we can order the Boolean functions that specify the genomes to be evolved by the decimal representation of their binary sequences. (B) Fitnesses of the possible binary genomes for 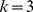 when 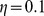 and 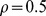. In this case, there are 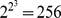 different rules of which 208 (81.1%) have a fitnesses of zero, while 47 (18.4%) have fitnesses greater than zero but smaller than 0.5. For 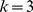, only Boolean function 232, the majority rule, has a large fitness. As 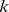 increases, the fraction of large fitnesses genomes decreases rapidly. (C) The total number of possible genomes grows super-exponentially with 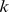. (D) The number of generations necessary for a classical genetic algorithm to find a large fitness genome also grows super-exponentially. This slow convergence is due to the fact the fraction of zero fitnesses genomes is growing with 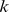. For this reason, it is not possible to use classical GAs to find solutions to complex problems when the fitness landscape is mostly flat.

Numerous studies have used GA to solve the density classification task [17]. The ''genetic materials'' being evolved in this case are one-dimensional Boolean functions (Fig. 1a). The fitness of a given genomic sequence is quantified by the encoded rule's efficiency in solving the density classification task. The smaller the difference 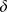 in the fraction of agents initially holding the majority and the minority states, the more challenging the task. Moreira *et al.* [16] showed that the majority rule with noisy communication and small-world connections among agents is able to solve the density classification task for 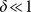 as long as the number of agents is large enough.

Moreira *et al.* [16] also found that, in order to efficiently complete the density classification task, the number of neighbors 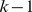 considered when using the majority rule must increase as the magnitude of the noise increases. Noise is implemented as follows: with probability 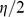 an input from a neighbor is replaced by the opposite value. That is, when noise acts an agent will perceive its neighbor to be in the opposite state. Note, however, that the noise does not change the state of the agents; agents always known their own state. If a certain agent is connected to 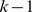 neighbors, noise may or may not act over each of these signals. Rules with 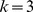 only perform efficiently if the communication noise is smaller than 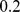, whereas if 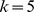, the majority rule is efficient for 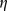 as large as 0.45. These results are consistent with the intuition that more complex rules, i.e. rules with more bits, are able to solve more challenging problems.

Navely, thus, it would seem to be a good strategy to start with a large genome allowing the GA to find the more robust solutions. However, as the number of bits increases, so does the size of the space of candidate solutions. While for rules considering the state of 2 neighbors there are 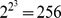 candidate solutions, for rules considering the state of 8 neighbors there are 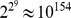. While for well-behaved fitness landscapes such a large space of candidate solutions is not a problem, for the density classification task the vast majority of candidate solutions have zero fitness, meaning that fitness selection is effectively blind (Fig. 1b).

The magnitude of the challenge is made clear in Figs. 1c and d. If the initial population of rules is chosen at random from the set of candidate solutions, then the number of generations necessary to obtain an efficient rule grows super-exponentially with 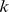. As a consequence, no efficient rule can be obtained for 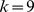 even after 10,000 generations of the GA.

### Variable Environment Genetic Algorithms

In order to solve problems characterized by golf-course-like fitness landscapes, we propose a schema that we denote *variable environment genetic algorithms* (VEGAs). The core of our approach is that simple versions of the problem of interest can be easily solved even if the fitness landscape is mostly flat and that the solutions to the simple problem can be used as stepping stones for finding more robust solutions able to solve more challenging problems.

**Figure 2:**
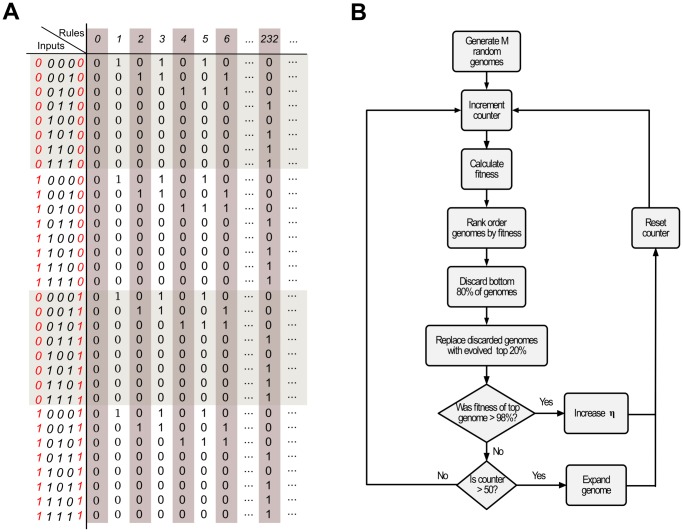
Implementing variable environment genetic algorithms. (A) To promote a rule with 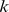 to 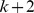, we include 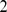 extra neighbors keeping the same output with any combination of these two new neighbors. On the left we see a possible input and its respective output for a 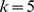 rule. On the right there are the respective possible outputs after generalization. Note that in the generalized rule the new inputs do not affect the rule's output. However, inter-generational mutation and crossover of the genetic material will yield changes in output that make the states of the new neighbors relevant. (B) Flowchart of the variable environment genetic algorithms. See the text for a more detailed description.

Our algorithm comprises two main stages (Fig. 2). The first stage, which we denote the generalization step, takes a selected genome with 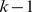 neighbors and creates a copy, but now with instructions for taking into consideration the information from two additional neighbors (see Fig. 2a for an example). Since the generalized genomes contain the ''base'' genomes, there is no evolutionary pressure to change any of its bits because they already have maximum fitness. After generalization of the selected genomes, we implement the second stage of the algorithm: We make the environment more demanding, creating an evolutionary pressure on the longer genomes.

### Simulation Details

We investigate systems with 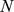 agents arranged in a ring and initially connected to their 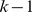 nearest neighbors. Next, we randomly select 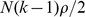 pairs of connections between agents and switch the end nodes of those connections, thus creating a small-world topology [18], [19].

For generation zero, we assign the same randomly-selected 32-bit (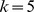) Boolean function — that is, the same genome — to each of the 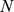 agents in a system. We repeat this step for each of the 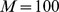 genomes in the population.

At time zero, we assign to each agent the majority state, 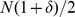 times, or the minority state, 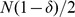 times. In each successive time step, all agents in a system interact synchronously according to the common Boolean function. Importantly, we implement communication noise in the connections between agents. Concretely, there is a probability 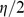 that an input from a neighbor is replaced by the opposite value. Note that the noise only affects the communication of the value of the state, not the actual state [16].

The density classification task is completed successfully if the states of *all* automata converge to the initial majority state — no partial credit is given. We define the fitness of a genome 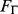 as the fraction of initial conditions with a given task difficulty 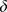 for which agents possessing genome 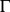 converge to the correct consensus within 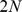 time steps. The introduction of noise in the dynamics results in a degree of randomness in the evolution of the interacting units. For this reason complete consensus may never emerge, therefore we assume that the task is completed successfully when, at the end of the evolution, any deviation from the correct classification is caused by these fluctuations, meaning that, if the noise were ''turned off'' at that moment, all the units would converge to the correct state in the next time step. This criteria works for finite systems, but if we considered 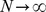 perfect consensus may never be achieved in a noisy environment. Moreira *et al* [16] showed that 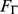 depends on the noise magnitude 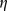, the rewiring probability 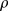, the number 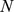 of agents in a system, and the task difficulty 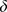.

We perform the density classification task 100 times for each genome with parameters 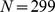, 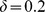, 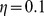, and 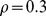. To assure an unbiased sampling of initial conditions, half of the realizations were started with 60% of the agents in state zero and the other half with 60% of the agents in state one. We define the fitness of a genome as the fraction of times that the system of agents sharing that genome converged to the correct consensus, that is, at the end of the process all agents hold the same state as the initial majority. Also, to reduce fluctuations, the set of initial conditions is the same for each genome, changing only after that generation step is completed.

After estimating their fitnesses, we rank the 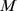 genomes and discard the bottom 80% of genomes. We then generate 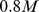 new genomes through crossover and mutation of randomly selected genomes from among the top 20% fittest genomes [1]. For 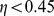, the GAs evolves genomes with fitnesses of nearly 100% after just a few tens of generations.

Whenever a genome with fitness greater than 0.98 evolves, we increase 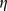 by 0.01, and re-estimate the fitness of all 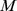 genomes in the population. Since there is a threshold value 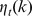 of the noise for which a genome with 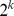 bits can have high fitness — for 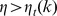 no fit genomes can evolve. Thus, if the GA cannot evolve an highly efficient genome after 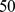 generations, we consider again the set of high fitness genomes that evolved for 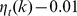 and generalize them (Fig. 2a).

The generalization step creates new genomes that are canalized [20] by the state of just 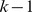 neighbors but that incorporate the ability to consider the state of two additional neighbors. After generalizing the genomes, we set 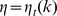 and use the traditional GA approach to evolve a high fitness genome (Fig. 2b).

## Results

**Figure 3:**
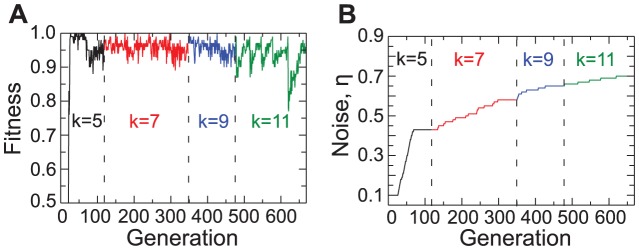
The Variable Environment Genetic Algorithm dramatically speeds up the evolution of solutions to the density classification task. (A) Highest fitness in the population and (B) noise magnitude 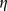 as a function generation number. We start simulations with 100 randomly selected populations of agents having genomes with 32 bits, that is, k = 5 and 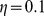. When a genome reaches a fitness of 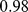, we increase the magnitude of the noise by 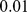. In the beginning of the process the most efficient rules are guessers, that have efficiency about 0.5. At some generation an innovative rule evolves that can classify both kinds of consensus, and in a few more generations the desired efficiency is achieved. The noise then increases rapidly until it reaches a critical level (about 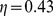). Then, no rule achieves the desired efficiency even after 50 generation. At this point, we promote the population of rules to include 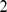 extra neighbors. The doted lines in the panels mark these moments. The promoted rules are essentially identical to the previous ones, with the extra neighbors acting as silent inputs, that is, the extra information does not affect the rule dynamics. The GA, by mutation and crossover, should make use of these new inputs to evolve more efficient rules. Eventually, members of the population will achieve the desired efficiency, noisy increases until it reaches another level that can not be surmounted by rules with this value of 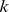. The rules are promoted again, and the process continues with noise and complexity of the rules co-evolving until we obtain highly complex rules (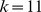) that sustain high noise levels (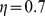).

We show in Fig. 3 the highest fitness in the population for 650 generations of the VEGAs process. While traditional GA would require about 3,000 generations to evolve a high fitness genome for 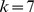, we see that already after only 130 generations of the VEGA process, we have evolved a highly fit genome with 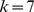. Remarkably, in only 600 generations, VEGA evolves a genome with 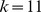 — that is, with 2048 bits — that displays high efficiency in solving the density classification task for 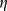 as high as 0.7. Extrapolating from the data in Fig. 1d, we would expect traditional GA to require on the order of 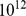 generations to accomplish the same.

**Figure 4:**
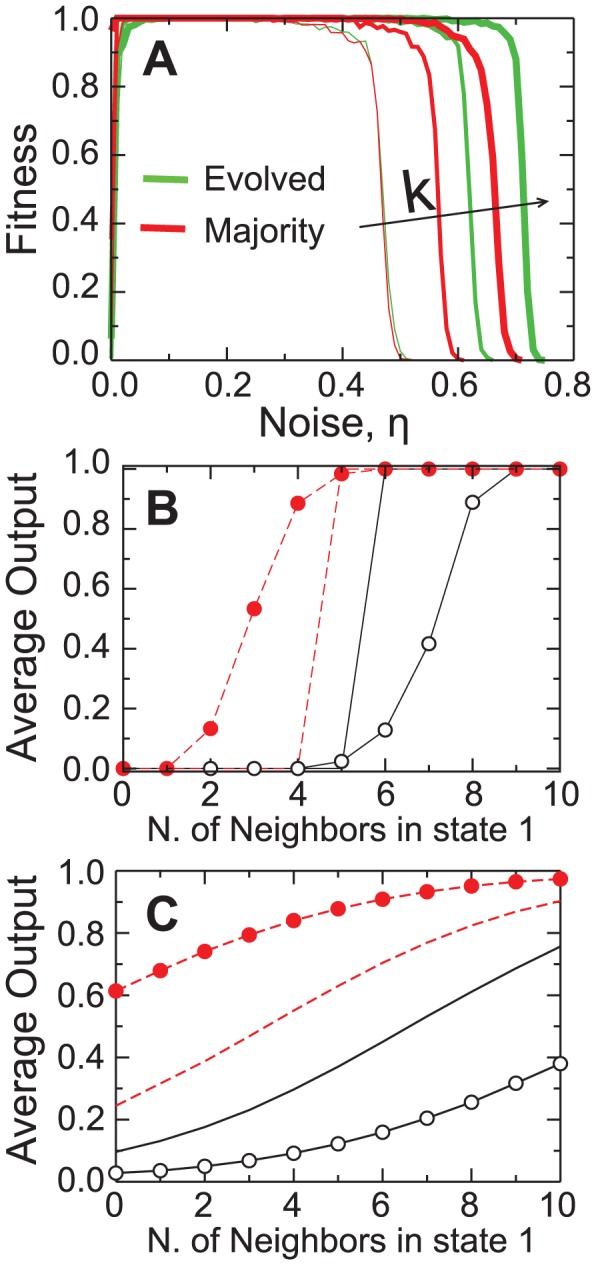
Robustness of the evolved genomes again communication noise. (A) Comparison of the fitness of the genomes evolved using VEGA against the fitness of the majority rule for 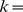
5, 7 and 11. It is visually apparent that for 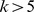 the evolved genomes are more robust against communication noise than the majority rule. (B) Average updated state of an agent with a high fitness evolved genome with 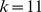 as a function of the number of neighbors in state one for 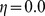. The red circles show the updated state of the agent when the initial state is one, and the red dashed line shows results for the majority rule for comparison. The empty circles show the updated state of the agent when the initial state is zero, and the black full line shows results for the majority rule. (C) Same as b, but for 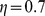. It is visually apparent that the evolved genomes are more conservative than the majority rule — they require larger majorities of neighbors on the opposite state in order to change state.

Interestingly, the evolved rule is more robust against communication noise than the majority rule (Fig. 4). This increased robustness recalls the findings of Seaver *et al.* [21] reporting that conservative strategies — that is, strategies that give greater weight to an agent's state than to the information received from the neighbors — display greater robustness again communication noise than the corresponding majority rule. In order to investigate whether the higher fitness of the evolved rules mirrors the conservative strategy discussed in Seaver *et al.* [21], we calculate the average updated state of an agent using a VEGA evolved genome as a function of the agent's initial state and of the number of neighbors in state one.

Since the evolved rules consider not only how many, but also which neighbors are in a given state, even without noise, the same number of neighbors in a given state can yield different outputs. For this reason, we obtain an average output of a class of inputs by counting the fraction of these outputs that turn the agent to state 1 for a noiseless system (Fig. 4b) and for 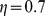 (Fig. 4c).

It is noteworthy that an agent's new state strongly depends on its prior state. When, for instance, the agent is initially in state 1, even for only 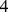 out of 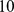 neighbors in state 1, it still keeps its initial state with approximately 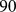
% of chance, while the majority rule would necessarily switch its state to conform with the majority. As a result, agents using the evolved rules tend to keep their own states, unless the overwhelming majority of their neighbors ''disagrees'' with them (Fig. 4b). These results show that to effectively perform the density classification task in noisy environments an agent becomes increasingly conservative, that is, it assigns greater importance to its own state than to the input from its neighbors.

## Discussion

Much of the literature on Monte Carlo sampling and optimization focuses on algorithms with improved performance in rugged landscapes. For instance, parallel tempering and simulated annealing algorithms draw inspiration from physics by introducing a ''temperature'' parameter. Higher temperature systems correspond to smoothed versions of the target landscape, in which exploration between multiple modes is facilitated [22], [23]. Other related methods belong to a broad class known as graduated optimization, in which solutions to simpler optimization problems define the starting point for progressively more difficult ones [24]. While smoothing is one powerful way to simplify the optimization, when the vast majority of the search space has zero gradient, smoothing will actually make the search more challenging. However, in some problems when exploration in high dimensional landscapes is intractable, optimization of sub-problems in a lower dimensional subspace is often feasible [25]. Likewise, the power of our method comes from the fact that we drastically reduce the initial size of this space of possible solutions, by simplifying the problem being solved.

Our study demonstrates that variable environment genetic algorithms have the potential to find very efficient solutions to complex problems characterized by flat fitness landscapes. While we consider, for illustration purposes, a case in which VEGAs increase task difficulty by controlling the noise level, one could easily generalize the process to cases in which the size of the system or the complexity of the interactions are increased.

Interestingly, our results may have some significance for the understanding of abiogenesis. One major open question in abiogenesis is how living cells arose from a concentrated soup of non-living complex organic polymers. A particularly challenging point in abiogenesis is to assign distinct fitnesses to different assemblies of organic polymers. In the absence of differences in fitness, life would have to have appeared as the result of random drift [26], [27], a possibility with a prohibitively low probability (as is exemplified by the data in Fig. 1). If an assembly of organic polymers is auto-catalytic, then it would be endowed with differential fitness based on how strong the reinforcement is. Such auto-catalytic networks would have their evolution driven by natural selection toward complexity and life. Our numerical results demonstrate that the solution of simple tasks that provide stepping-stones toward the solution of an ''impossibly'' complex problem can, in fact, lead to reductions by tens of orders of magnitude of the time needed to find efficient solutions.

Our study also provides support for the natural emergence of conservative strategies as a response to the challenge of information integration in complex environments. Many experiments show that individuals easily fall prey to peer pressure and change their opinion to conform to the majority view. But some studies also show that conservatism is a widespread human tendency. For example, in the Asch conformity experiments [28], the number of opposing opinions necessary to change an individual belief is always close to the total number of actors. Being conservative, therefore, may act as an efficient countermeasure to noisy information and scenarios where neighbors' opinions are not necessarily reliable.
